# Significance of Concurrent Chemoradiotherapy as Primary Treatment in Patients with Metastatic Cervical Cancer

**DOI:** 10.3390/curroncol28030155

**Published:** 2021-04-29

**Authors:** Satomi Hattori, Nobuhisa Yoshikawa, Kazumasa Mogi, Kosuke Yoshida, Masato Yoshihara, Satoshi Tamauchi, Yoshiki Ikeda, Akira Yokoi, Kimihiro Nishino, Kaoru Niimi, Shiro Suzuki, Hiroaki Kajiyama

**Affiliations:** 1 Department of Obstetrics and Gynecology, Nagoya University Graduate School of Medicine, Tsurumai-cho, Showa-ku, Nagoya 466-8550, Japan; shattori33@med.nagoya-u.ac.jp (S.H.); mogi.kazumasa@med.nagoya-u.ac.jp (K.M.); yoshida.kousuke@med.nagoya-u.ac.jp (K.Y.); myoshihara1209@med.nagoya-u.ac.jp (M.Y.); tama-sato@med.nagoya-u.ac.jp (S.T.); ikeda-yoshiki@med.nagoya-u.ac.jp (Y.I.); ayokoi@med.nagoya-u.ac.jp (A.Y.); kimikimi@med.nagoya-u.ac.jp (K.N.); kaorun@med.nagoya-u.ac.jp (K.N.); kajiyama@med.nagoya-u.ac.jp (H.K.); 2 Department of Gynecologic Oncology, Aichi Cancer Center Hospital, Nagoya 464-0021, Japan; s.suzuki@aichi-cc.jp

**Keywords:** metastatic cervical cancer, tumor size, concurrent chemoradiotherapy, biomarker, prognosis

## Abstract

(1) This study investigated the prognostic impact of tumor size in patients with metastatic cervical cancer. (2) Methods: Seventy-three cervical cancer patients in our institute were stratified into two groups based on distant metastasis: para-aortic lymph node metastasis alone (IIIC2) or spread to distant visceral organs with or without para-aortic lymph node metastasis (IVB) to identify primary tumor size and concurrent chemoradiotherapy. (3) Results: The overall survival (OS) for patients with a tumor >6.9 cm in size was significantly poorer than that for patients with a tumor ≤6.9 cm in the IVB group (*p* = 0.0028); the corresponding five-year OS rates in patients with a tumor ≤6.9 and >6.9 cm were 53.3% and 13.4%, respectively. In the multivariate analysis, tumor size and primary treatment were significantly associated with survival in metastatic cervical cancer. (4) Conclusions: Tumor size ≤6.9 cm and concurrent chemoradiotherapy as the primary treatment were favorable prognostic factors for patients with metastatic cervical cancer.

## 1. Introduction

In 2018, approximately 570,000 cervical cancer cases were diagnosed worldwide, and over 310,000 people died from disease progression [1]. Although cervical cancer remains an important health issue, the incidence of cervical cancer and precancerous cervical lesions has successfully decreased as a result of efforts to prevent onset and early detection by the adoption of human papilloma virus vaccines and cervical cancer screenings [2,3]. Conversely, in Japan, the number of cervical cancer cases has increased each year in recent years, and the number of deaths has not decreased because of the low screening rate and vaccine avoidance [4]. Thus, cervical cancer should still be recognized as a fatal disease, and many patients often display an advanced stage with distant metastases at diagnosis.

For advanced cervical cancer with distant metastases, treatment with concurrent chemoradiotherapy (CCRT), systemic chemotherapy, or radiation therapy (RT) is normally performed. Although CCRT is principally recommended only for patients with locally advanced cervical cancer, it is also applied for patients with advanced cervical cancer with para-aortic lymph node (PAN) metastasis, which can be curable [5]. Because metastatic cervical cancer with hematogenous metastasis such as liver metastasis and lung metastasis is rarely curable, RT alone, systemic chemotherapy, and palliative care are often options rather than CCRT [5]. However, for some recurrent metastatic cervical cancer cases with PAN metastasis alone, long-term survival has been achieved [6,7]. Therefore, the metastatic cervical cancer patient population is heterogeneous and includes patients with different types of metastases, such as lymphatic metastases, hematogenous metastases, and peritoneal metastases. In fact, in the new staging of cervical cancer (International Federation of Obstetrics and Gynecology (FIGO) 2018), PAN metastasis was clearly classified as stage IIIC2, and metastasis to other distant organs was clearly classified as stage IVB [8]. To select the most optimal treatment for patients with metastatic cervical cancer, a robust prognostic marker that can visualize the course of treatment based on the patient’s condition must be identified.

Clinical biomarkers to predict survival outcomes have been widely investigated in the field of cervical cancer. Patient age, performance status, tumor size, and lymph node status were significantly associated with progression-free survival (PFS) in patients with locally advanced cervical cancer treated by RT [9]. A recent study reviewing over 2000 patients with locally advanced cervical cancer enrolled in Gynecologic Oncology Group clinical trials of radiotherapy and concurrent chemoradiotherapy demonstrated that histology, race, performance status, tumor size, FIGO stage, tumor grade, pelvic node status, and treatment with concurrent cisplatin-based chemotherapy were associated with PFS and overall survival (OS) [10]. In a few studies evaluating the prognostic factors of metastatic cervical cancer, the site of metastasis, performance status, and histology have been reported as significant predictors of survival [7,11,12]. To our best knowledge, there are currently few reports demonstrating the significance of tumor size in metastatic cervical cancer.

This study was undertaken to identify the prognostic significance of tumor size in patients with metastatic cervical cancer.

## 2. Materials and Methods

We retrospectively analyzed the medical records of cervical cancer patients who were pathologically diagnosed in our institute from December 2004 to December 2017. This study was approved by the Institutional Review Board (IRB) (approval number 2019-0106). For this study, the IRB issued a waiver for written informed consent because data collection was retrospective. All methods involving the human participants were carried out in accordance with the ethical principles of the Declaration of Helsinki.

Of the 838 patients in total, 75 (8.9%) were diagnosed with primary cervical cancer with distant metastases by physical examination and/or diagnostic computed tomography (CT) imaging. Two cases with insufficient data were excluded; thus, 73 people were finally included in the analysis. Patients with para-aortic lymph node metastasis alone were included. We extracted factors that may affect survival outcome, such as age, preoperative body mass index (BMI), histological type, TNM classification, tumor diameter in the greatest dimension, preoperative value of the tumor marker, and modality of primary treatment, from the patients’ medical records. Tumor diameter in the greatest dimension was determined as a value of long diameter in T2-weighted images in the sagittal plane.

The treatment for each patient was determined by the discussion of several gynecologic oncologists and radiologists in our hospital. Our group principally selected CCRT as the primary treatment for the control of local tumors even in patients with metastatic cervical cancer. Surgery, radiation alone, or systemic chemotherapy were performed when the patients desired treatment other than CCRT, or in situations where treatment options other than CCRT were desirable because of severe renal dysfunction or low performance status. CCRT principally consisted of external beam radiotherapy (EBRT) and intracavitary brachytherapy (ICBT) with concurrent chemotherapy. In EBRT, whole-pelvic irradiation was administered to all patients, and subsequent para-aortic irradiation was added in principle for patients with PAN metastasis. The standard dose of the whole-pelvic irradiation and para-aortic irradiation was 50.4 Gy and 46 Gy, respectively, which was delivered in 1.8–2.0 Gy per dose five days a week. In patients whose primary tumors were well controlled after 36–39.6 Gy of whole pelvic EBRT, intracavitary brachytherapy (ICBT) using a remote after-loading system of a Co60 source was administered with central shielding EBRT. The total dose to point A (a reference location 2 cm lateral and 2 cm superior to the cervical os) was 18–24 Gy. Concurrent chemotherapy consisted of three to five cycles of 5-fluorouracil (700 mg/m^2^ for four consecutive days)/cisplatin (70 mg/m^2^ on the first day) every three weeks. After primary CCRT, some patients received subsequent systemic chemotherapy, which consisted of paclitaxel (180 mg/m^2^) and carboplatin (area under the curve (AUC = 5–6) every three weeks, paclitaxel (180 mg/m^2^) and carboplatin (AUC = 6) plus bevacizumab (15 mg/m^2^) every three weeks, carboplatin (AUC = 6) monotherapy every three weeks, and 5-fluorouracil oral therapy. Other treatment options were RT alone, radical hysterectomy followed by adjuvant CCRT or chemotherapy, primary systemic chemotherapy, and palliative therapy. Primary systemic chemotherapy consisted of paclitaxel (180 mg/m^2^) and carboplatin (AUC = 6) every three weeks. Bevacizumab (15 mg/m^2^) was administered to two patients with paclitaxel and carboplatin.

Post-treatment follow-up was conducted monthly for the first year, and the interval was extended after the second year. Regular follow-up was based on physical examination, transvaginal ultrasound, and blood tests, including tumor markers to detect disease progression. CT was performed at least every three to six months. 

Two parameters, the OS and PFS, were analyzed. OS was defined as the time between the beginning of primary therapy and death by any cause. PFS was defined as the time between the beginning of primary therapy and tumor progression, relapse, or death by any cause. 

Statistical analyses were performed with JMP 14.0 (SAS Institute Inc., Cary, NC, USA). A receiver operating characteristic (ROC) curve analysis of one-year survival according to overall survival one year after the beginning of primary treatment was performed to determine the optimal cutoff value, which was determined as the value of the area under the ROC curve. The patient characteristics were compared using the qualitative chi-square test and the quantitative Mann–Whitney U test. The Kaplan–Meier method was performed to analyze survival. Moreover, p-values were calculated with the log-rank test. To minimize confounding bias, a multivariate analysis was performed using the Cox proportional hazards model. A *p*-value of <0.05 represented statistical significance.

## 3. Results

A total of 73 patients were stratified into two groups based on the status of their distant metastasis: PAN metastasis alone (IIIC2, *n* = 28) or spread to distant organs or distant lymph node with or without PAN metastasis (IVB, *n* = 45). The clinicopathologic characteristics of all patients stratified into the two groups are shown in Table 1. Although age at diagnosis, preoperative body mass index (BMI), histological type, squamous cell carcinoma (SCC) antigen value, CA125 value, and primary treatment were similar between the two groups, the severity of tumor invasion, the involvement of the pelvic lymph node, and the tumor diameter in the greatest dimension were significantly more severe in the IVB group than in the IIIC2 group. Three of twenty-three (13.0%) patients in the IIIC2 group and 15 of 32 (46.9%) patients in the IVB group received subsequent systemic chemotherapy. Subsequent systemic chemotherapy consisted of paclitaxel and carboplatin in 14 patients, paclitaxel and carboplatin plus bevacizumab in 2 patients, carboplatin monotherapy in 1 patient, and 5-fluorouracil oral therapy in 1 patient. Fifteen of twenty-three (65.2%) patients in the IIIC2 group, who had complete remission of their disease after primary CCRT, and 9 of 32 (28.1%) patients in the IVB group, who had partial or complete remission, were followed up without additional treatment. In the IVB group, sites of distant metastasis are shown in Supplementary Table S1. With respect to tumor local control, 15 of 23 (65.2%) patients in the IIIC2 group had complete remission of the primary tumor after CCRT, and 12 of 32 (37.5%) patients in the IVB group had complete remission. Furthermore, 3 of 23 (13.0%) patients in the IIIC2 group had partial response of the primary tumor, and 16 of 32 (50.0%) patients in the IVB group had partial response. ICBT was delivered in 18 of 23 patients in stage IIIC2 and 23 of 32 patients in stage IVB.

**Figure 1:**
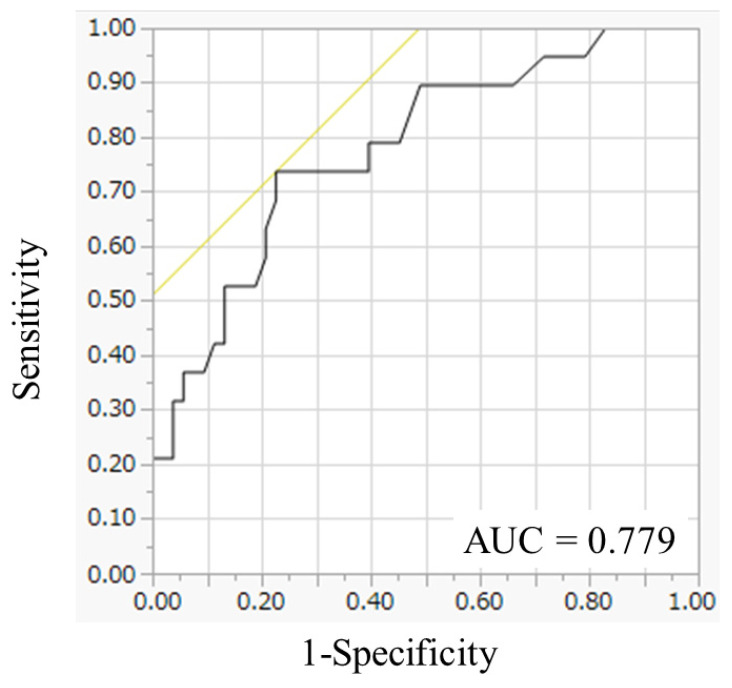
Receiver operating characteristic curve analysis of tumor diameter in the greatest dimension. The area under the curve for tumor diameter in the greatest dimension was 0.779.

Next, to identify the optimal value for predicting survival, an ROC curve for one-year survival was generated. The area under the curve for tumor diameter in the greatest dimension was 0.779, and the optimal cutoff value for predicting one-year survival was 6.9 cm (Figure 1). The determined optimal cutoff value for tumor diameter in the greatest dimension had a 73.6% sensitivity and a 77.3% specificity.

Table 2 classifies the patients by clinical tumor size at diagnosis. The mean and median tumor size for all 73 patients was 6.44 cm and 5.85cm, respectively (95% confidential interval (CI): 5.88–7.00, range: 3.0–14.2). Patients in the IVB group had a significantly larger tumor size than patients in the IIIC2 group (mean 6.94 vs. 5.66 cm (*p* = 0.0266)). Classification by category using the cutoff value (6 cm) also showed a significant difference in tumor size between the two groups (*p* = 0.0081).

The upper row of Table 3 shows the results of the univariate analysis of clinicopathologic variables that are potentially related to survival. For all 73 patients, tumor size >5 cm (hazard ration (HR) = 2.19, 95% confidence interval (CI): 1.07–4.92; *p* = 0.0291), tumor size > 6 cm (HR = 2.19, 95% CI: 1.15–4.25; *p* = 0.0163), tumor size > 6.9 cm (HR = 2.48, 95% CI: 1.30–4.73; *p* = 0.006), and primary treatment other than CCRT (HR = 3.69, 95% CI: 1.69–7.57; *p* = 0.0015) were significantly associated with a shorter OS. For patients in the IIIC2 group, although a preoperative BMI > 22 (HR = 0.29, 95% CI: 0.06–0.97; *p* = 0.0444) was significantly related to a favorable prognosis, primary treatment other than CCRT (HR = 10.98, 95% CI: 2.11–80.01; *p* = 0.0049) was a statistically significant prognostic factor for a shorter OS. For the patients in the IVB group, tumor size > 6.9 cm (HR = 3.47, 95% CI: 1.53–8.33; *p* = 0.0028) and primary treatment other than CCRT (HR = 3.12, 95% CI: 1.17–7.51; *p* = 0.024) were statistically significant prognostic factors of a shorter OS. The lower row of Table 3 presents the multivariate analysis. Because >6.9 cm was the optimal cutoff to predict shorter OS in both the IIIC2 and IVB groups, tumor size (>6.9 vs. ≤6.9 cm) was also included as a variable in the multivariate analysis, as were age at diagnosis, preoperative BMI, histological type, clinical tumor staging, status of lymph node metastasis, SCC, CA125, and primary treatment. The Cox proportional hazard models revealed that a tumor size > 6.9 cm for all 73 patients (HR = 3.54, 95% CI: 1.67–7.68; *p* = 0.001) and for patients in the IVB group (HR = 4.79, 95% CI: 1.83–14.16; *p* = 0.0012) was significantly associated with a poorer prognosis than a tumor size ≤ 6.9 cm. Furthermore, CCRT as the primary treatment for all 73 patients (HR = 3.80, 95% CI: 1.55–9.14; *p* = 0.0039) and for patients in the IVB group (HR = 5.24, 95% CI: 1.65–16.59; *p* = 0.0056) was significantly associated with a more favorable prognosis than other therapies as the primary treatment. 

**Figure 2:**
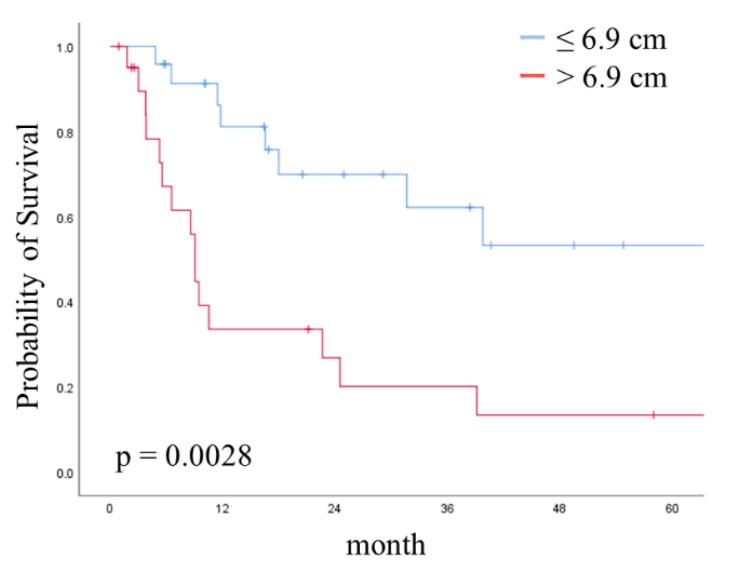
Kaplan–Meier curve showing overall survival (OS) stratified by tumor size in the IVB group. Curves show OS stratified by tumor size at the cut-off of 6.9 cm. *p*-value was calculated with the log-rank test.

Finally, to evaluate the prognostic significance of tumor diameter in the greatest dimension, we performed a survival analysis. In the Kaplan–Meier analysis, the five-year OS and PFS rates in all patients were 37.4% and 16.8%, respectively. Although the OS in the IIIC2 group was longer than that in the IVB group, the difference was not statistically significant (*p* = 0.1115). Conversely, in a comparison of groups stratified by a tumor diameter of 6.9 cm in the greatest dimension, the >6.9 cm group exhibited a significantly shorter OS than the ≤6.9 cm group for all patients (*p* = 0.0038). Although the OS for patients whose tumor size was ≤6.9 cm was not significantly better than that for patients whose tumor size was >6.9 cm in the IIIC2 group (*p* = 0.7443), the OS for patients with a tumor greater than 6.9 cm in size was significantly poorer than that for patients with a tumor ≤6.9 cm in the IVB group (*p* = 0.0028); the corresponding five-year OS rates in patients with a tumor size ≤6.9 cm and >6.9 cm were 53.3% and 13.4%, respectively (Figure 2).

## 4. Discussion

The precise staging of cervical cancer is essential for determining the optimal treatment strategy. The FIGO staging system is clinically well-accepted for selecting appropriate treatment for patients with cervical cancer and for predicting the prognosis for cervical cancer. For metastatic cervical cancer belonging to FIGO 2018 stage IIIC2 or IVB, various treatments such as CCRT, systemic chemotherapy, palliative RT, and best supportive care can be options. However, there are limited reports of prognostic factors that can predict cases of metastatic cervical cancer; thus, treatment options are generally selected based on performance status. This retrospective study identified the prognostic impact of local tumor size and the significance of CCRT in patients with metastatic cervical cancer. 

There have been many reports on the correlation between primary tumor size and prognosis in early cervical cancer. Previous reports have shown that tumor size is an independent prognostic factor in patients with stage II cervical cancer (1988/2002 FIGO staging system) [13,14]. Kato et al. reported that patients with a tumor ≤2 cm had a more favorable OS than patients with a tumor > 2 cm in stage IB1 cervical cancer (2009 FIGO staging system) [15]. Furthermore, Wagner et al. investigated the impact of tumor size in stage I-IIIB cervical cancer by analyzing the Surveillance, Epidemiology, and End Results database and demonstrated that size is an independent prognostic factor across stages I-IIIB, which validates the update of the FIGO staging system in 2009 [16]. In the latest 2018 FIGO staging system, stage IB disease from the 2014 FIGO staging system was reclassified into three groups according to tumor size: stage IB1 (<2 cm), stage IB2 disease (2–3.9 cm), and stage IB3 (≥4 cm) [17]. Thus, it is clear that the importance of tumor size has come to be reevaluated based on the revision of the FIGO staging system, which is indispensable for selecting treatment options.

Our results demonstrated that the primary tumor size of patients with distant metastases has a significant impact on long-term prognosis. In the IVB group in particular, a statistically significant improvement in OS was found for patients with a tumor size >6.9 cm compared to ≤6.9 cm. Prognostic factors for metastatic cervical cancer have been previously investigated and have indicated that larger tumor size, poorer performance status (PS), older age, histological subtype of adenocarcinoma, and presence of main organ metastases were associated with survival [11,18,19,20]. A retrospective analysis of 50 patients with metastatic cervical cancer identified a significant correlation between tumor size (cutoff: 4 cm) and survival [20]. Conversely, Nishio et al. (*n* = 36) and Kim et al. (*n* = 30) reported that there were no significant associations between tumor size and survival [18]. Although the significance of tumor size in metastatic cervical cancer remains controversial, it could be better assessed by optimizing the tumor size cutoff and increasing the number of patients in this study (*n* = 73). 

We noted that patients receiving CCRT as their primary treatment had a more favorable prognosis compared to other treatments. Although CCRT is the standard therapy for locally advanced cervical cancer patients, CCRT is not principally recommended for patients with metastatic cervical cancer, particularly for those with distant visceral organ metastases [5]. However, treatment for metastatic cervical cancer should be flexible, as neo-adjuvant chemotherapy followed by radical surgery can be an option as well as primary CCRT for locally advanced cervical cancer [21]. In patients with metastatic cervical cancer, the primary lesion is often bulky, which results in not only shortening the prognosis but also reducing quality of life (QOL) and PS. Proactive CCRT for patients with metastatic cervical cancer that provides good local control has led to the expansion of treatment options for metastatic lesions through improvements in QOL and PS, which may have contributed to improved prognosis.

There are several limitations in this study due to its retrospective nature and long duration. It was difficult to refer to an accurate PS from the medical records, so PS was excluded from this analysis. PS is an important status that reflects the general condition and self-care ability of patients, and it has been reported to be correlated with prognosis in many malignant tumors [22,23,24]. Presumably, since tumor growth worsens PS, tumor size, which was an independent prognostic factor in this study, may be correlated with PS. Furthermore, with respect to the prognostic impact of CCRT, there may be a selection bias in which CCRT is more likely to be selected in patients with good PS. CCRT is not recommended for all patients with metastatic cervical cancer, although there is no doubt that there are patients with long-term survival with CCRT. In addition, due to the small number of patients treated with bevacizumab in this study, it was not possible to compare the efficacy of CCRT with that of combination chemotherapy including bevacizumab. Recently, our policy has been to choose CCRT or combination therapy including bevacizumab based on the status of the primary tumor, metastases, and general condition. This study is a hypothesis-generating study that demonstrated the prognostic impact of tumor size and CCRT in metastatic cervical cancer.

## 5. Conclusions

In conclusion, a tumor size ≤ 6.9 cm and CCRT were favorable prognostic factors for patients with metastatic cervical cancer, especially in stage IVB.

## Figures and Tables

**Table 1:** Patients’ clinical characteristics stratified by the IIIC2 and IVB groups.

Variable	All Cases (*n* = 73)		IIIC2 Group (*n* = 28)		IVB Group (*n* = 45)		
	*n*	%	*n*	%	*n*	%	*p*-Value
**Age, years**							
mean ± SD	55.6 ± 12.6		53.3 ± 11.8		57.0 ± 13.1		0.2363
≤55	34	46.6	16	57.1	18	40.0	0.1529
>55	39	53.4	12	42.9	27	60.0	
**Preoperative BMI**							
mean ± SD	20.8 ± 3.6		21.3 ± 3.8		20.5 ± 3.5		0.3929
≤22	50	68.5	20	71.4	30	66.7	0.6691
>22	23	31.5	8	28.6	15	33.3	
**Histology**							
SCC	49	67.1	19	67.8	30	66.6	0.5124
AC	19	26.0	6	21.4	13	28.8	
Others	5	6.8	3	10.7	2	4.4	
**TNM classification**							
**cT**							
1	7	9.5	1	3.6	6	13.3	0.0021
2	32	43.8	20	71.4	12	26.7	
3	22	30.1	6	21.4	16	35.6	
4	11	15.0	1	3.6	9	20.0	
NA	1	1.3	0	0.0	2	4.4	
**N**							
0	8	10.9	0	0.0	8	17.8	0.00035
1	64	87.6	28	100.0	36	80.0	
NA	1	1.3	0	0.0	1	2.2	
**Tumor size, mm**							
mean ± SD	64.4 ± 23.9		56.6 ± 4.4		69.4 ± 3.5		0.0266
≤69	45	61.6	21	75.0	24	53.3	0.0603
>69	28	38.4	7	25.0	21	46.7	
**SCC antigen value, ng/mL**							
median (range)	14.4 (0.6–465.6)		15.4 (1–349.6)		11.5 (0.6–465.6)		
≤10 or NA	37	50.7	13	46.4	24	53.3	0.566
>10	36	49.3	15	53.6	21	46.7	
**CA125, U/mL**							
median (range)	59.4 (3.4–19761)		84.7 (3.4–1018)		48.3 (9.3–19761)		
≤100 or NA	51	69.9	19	67.9	32	71.1	0.7688
>100	22	30.1	9	32.1	13	28.9	
**Primary Treatment**							
CCRT	55	75.3	23	82.1	32	71.1	0.28
Others	18	24.7	5	17.9	13	28.9	
Surgery	4	(22.2)	3	(60.0)	1	(7.7)	
Chemotherapy	1	(5.6)	0	(0.0)	1	(7.7)	
Radiation	13	(72.2)	2	(40.0)	11	(84.6)	

SD, standard deviation; AC, adenocarcinoma; SCC, squamous cell carcinoma; NA, not available; CCRT, concurrent chemoradiotherapy.

**Table 2:** Tumor size distributions.

Tumor Size (cm) at Diagnosis	All Cases (*n* = 73)		IIIC2 Group (*n* = 28)		IVB Group (*n* = 45)		*p*-Value
	*n*	%	*n*	%	*n*	%	
≤4	9	12.3	4	14.3	5	11.1	0.6904
>4	64	87.6	24	85.7	40	88.9	
≤5	24	32.8	13	46.4	11	24.4	0.0533
>5	49	67.1	15	53.6	34	75.6	
≤6	38	52.1	20	71.4	18	40.0	0.0081
>6	35	47.9	8	28.6	27	60.0	
≤6.9	45	61.6	21	75.0	24	53.3	0.0603
>6.9	28	38.4	7	25.0	21	46.7	
Mean (SD)	6.44 (5.88–7.00)		5.66 (4.98–6.34)		6.94 (6.14–7.74)		0.0266
Median (range)	5.85 (3.0–14.2)		5.20 (3.0–10.4)		6.55 (3.1–14.2)		

SD, standard deviation.

**Table 3:** Cox regression models for overall survival.

Variable	Category	All Patients	*p*-Value	IIIC2 Group	*p*-Value	IVB Group	*p*-Value
		HR (95% CI)		HR (95% CI)		HR (95% CI)	
**Univariate Analysis**							
Tumor size (cm)	>4 vs. ≤4	1.35 (0.53–4.54)	0.5539				
	>5 vs. ≤ 5	2.19 (1.07–4.92)	0.0291				
	>6 vs. ≤6	2.19 (1.15–4.25)	0.0163				
	>6.9 vs. ≤6.9	2.48 (1.30–4.73)	0.006	1.21 (0.33–3.66)	0.7443	3.47 (1.53–8.33)	0.0028
Metastatic status	IVB vs. IIIC2	1.69 (0.88–3.38)	0.1115	NA	NA	NA	NA
Age at diagnosis	>55 vs. ≤55	0.96 (0.50–1.83)	0.9178	0.46 (0.12–1.40)	0.1785	1.34 (0.58–3.26)	0.4857
Preoperative BMI	>22 vs. ≤22	0.65 (0.29– 1.30)	0.2369	0.29 (0.06–0.97)	0.0444	0.99 (0.38–2.31)	0.9935
Histology	AC or others vs. SCC	1.40 (0.70–2.68)	0.3282	2.51 (0.85–7.42)	0.0921	0.90 (0.34–2.10)	0.8238
cT	3–4 vs. 1–2	1.35 (0.70–2.57)	0.3547	0.91 (0.20–2.95)	0.8904	1.38 (0.61–3.23)	0.4381
N	1 vs. 0 or NA	0.89 (0.40–2.38)	0.8118	NA	NA	1.15 (0.48–3.19)	0.7587
SCC	>10 vs. ≤10	1.21 (0.64–2.31)	0.5433	0.84 (0.27–2.44)	0.7594	1.67 (0.73–3.92)	0.2194
CA125	>100 vs. ≤100	0.90 (0.40–1.86)	0.8021	1.49 (0.45–4.35)	0.4868	0.64 (0.18–1.72)	0.4017
Primary Treatment	Others vs. CCRT	3.69 (1.69–7.57)	0.0015	10.98 (2.11–80.01)	0.0049	3.12 (1.17–7.51)	0.024
**Multivariate Analysis**							
Tumor size	>6.9 vs. ≤6.9	3.54 (1.67–7.68)	0.001	4.90 (0.69–37.80)	0.1079	4.79 (1.83–14.16)	0.0012
Metastatic status	Distant vs. PaLN only	1.87 (0.87–4.06)	0.1036	NA	NA	NA	NA
Age at diagnosis	>55 vs. ≤55	1.08 (0.50–2.27)	0.8279	0.10 (0.007–0.85)	0.0345	1.63 (0.62– 4.40)	0.3143
Preoperative BMI	>22 vs. ≤22	0.53 (0.21–1.19)	0.1297	0.11 (0.008–0.80)	0.0274	0.63 (0.20– 1.73)	0.3806
Histology	AC or others vs. SCC	2.01 (0.73–5.75)	0.174	4.88 (0.82–41.21)	0.0818	1.48 (0.38– 6.00)	0.5687
cT	3–4 vs. 1–2	0.95 (0.40–2.22)	0.9201	3.09 (0.38–25.21)	0.2738	1.12 (0.38– 3.33)	0.8315
N	1 vs. 0 or NA	1.56 (0.58–4.74)	0.3795	NA	NA	1.32 (0.48– 4.16)	0.5999
SCC	>10 vs. ≤10	1.49 (0.63–3.71)	0.3659	7.28 (0.80–201.09)	0.1038	1.41 (0.44– 4.95)	0.5636
CA125	>100 vs. ≤100	0.87 (0.33–2.10)	0.7695	0.36 (0.07–1.54)	0.1713	0.97 (0.25– 3.11)	0.9654
Primary Treatment	Others vs. CCRT	3.80 (1.55–9.14)	0.0039	2.95 (0.36–28.34)	0.3053	5.24 (1.65– 16.59)	0.0056

BMI, body mass index; NA, not available; AC, adenocarcinoma; SCC, squamous cell carcinoma; NA, not available; CCRT, concurrent chemoradiotherapy; HR, hazard ration; CI, confidence interval.

## Data Availability

Data sharing is not applicable to this article due to ethical restrictions.

## Supplementary Materials

The following are available online at https://www.mdpi.com/article/10.3390/curroncol28030155/s1. Table S1. Patients’ characteristics regarding metastasis in stage IVB group.
