# Supplementary material for: Significance of Concurrent Chemoradiotherapy as Primary Treatment in Patients with Metastatic Cervical Cancer
Source: Curr Oncol. 2021 Apr 29;28(3):1663–72. doi: 10.3390/curroncol28030155 (PMC8161771; doi:10.3390/curroncol28030155)
Supplement: Supplementary file 1 [file curroncol-28-00155-s001.zip › curroncol-1168815-supplementary.pdf]

Supplementary Materials

Table S1. Patients' characteristics regarding metastasis in stage IVB group.

Site of Distant Metastasis	n	%
DLN only	6	13.3
PaLN + DLN	4	8.9
Distant organ only	22	48.9
Lung	12	(54.5)
Bone	2	(9.1)
Liver	1	(4.5)
Peritoneum	3	(13.6)
Lung + Bone	1	(4.5)
Liver + Bone	2	(9.1)
Liver + Peritoneum	1	(4.5)
Distant organ + PaLN	13	28.9
Lung + PaLN	4	(30.8)
Liver + PaLN	1	(7.7)
Peritoneum + PaLN	2	(15.4)
Rectum + PaLN	1	(7.7)
Lung + Liver + PaLN	3	(23.1)
Lung + Bone + PaLN	1	(7.7)
Bone + Peritoneum + PaLN	1	(7.7)

PaLN, paraaortic lymph node; DLN, distant lymph node.
